# Influence of COVID-19 pandemic on hospitalisations at a paediatric traumatology department during 2020: a single-centre observational study and comprehensive literature review

**DOI:** 10.1007/s00068-024-02453-7

**Published:** 2024-01-30

**Authors:** Heide Delbrück, Ellen Lambertz, Filippo Migliorini, Nina Berger, Frank Hildebrand

**Affiliations:** 1 Department of Orthopaedics, Trauma and Reconstructive Surgery, University Hospital RWTH Aachen, Pauwelsstrasse 30, 52074 Aachen, Germany; 2 Department of Orthopaedic and Trauma Surgery, Academic Hospital of Bolzano (SABES-ASDAA), Teaching Hospital of the Paracelsus Medical University, 39100 Bolzano, Italy; 3 Department of Neuromuscular and Paediatric Orthopaedics, Klinikum Dritter Orden München – Nymphenburg, Menzinger Strasse 44, 80638 München, Germany

**Keywords:** COVID, Pandemic, Children, Paediatric, Injury, Trauma

## Abstract

**Purpose:**

The study investigates changes in the injury characteristics of hospitalised children in a paediatric trauma centre during the COVID-19 pandemic.

**Methods:**

Data from injured children from the pre-pandemic year 2019 were compared to the pandemic year 2020 using Pearson’s chi-squared test and the Mann–Whitney U test. The period of highly restrictive regulations (HRP) was evaluated separately. A comprehensive literature review with defined search terms resulted in a descriptive data synthesis.

**Results:**

Data from 865 patients indicated reductions in admissions of 5.6% and 54.4% during the HRP. In 2020, the hospitalisation time was longer (2.2 ± 2.7 days in 2019 vs. 2.4 ± 2.6 in 2020, *p* = 0.045); the proportions of wounds requiring surgical therapy (*p* = 0.008) and of observational treatments, primarily for mild brain injuries (*p* = 0.046), were higher; and conservative treatments, primarily for contusions, were lower (*p* = 0.005). There were no significant changes in age, location of lesions, or frequency of surgical therapy; nor were there differences in the HRP, except for fewer injuries in school and kindergarten (*p* < 0.001). The literature review summarises the main results of 79 studies.

**Conclusion:**

Limited resources did not alter the indications for surgical therapy. Further studies should examine whether the more common injuries sustained at home were caused by excessive work/childcare demands on parents. Reduced inpatient conservative treatment implies that hospital resources possibly were overused previously. The literature offers answers to many detailed questions regarding childhood injuries during a pandemic and more efficient safe treatment.

**Registration** Ethical committee of RWTH Aachen University EK 22-320; Center for Translational & Clinical Research RWTH Aachen University (CTC-A) 21-430.

**Supplementary information:**

The online version contains supplementary material available at 10.1007/s00068-024-02453-7.

## Introduction

On March 11, 2020, the World Health Organization (WHO) declared COVID-19 a worldwide pandemic. The subsequent lockdown in 2020 resulted in a significant change in the locations and activities of children and adolescents compared to 2019. Therefore, it seems likely that injury patterns and frequencies among children also changed during this period. Many international studies have focused on the influence of the lockdown on the characteristics of childhood injuries and changes in their management during this time [1–31]. However, to the best of our knowledge, no study has reported data from Germany regarding these aspects. Therefore, we aimed to report changes in admissions to the paediatric trauma centre of our university hospital in North Rhine-Westphalia/Germany as well as alterations in injury patterns and treatments.

We performed a comprehensive systematic literature search to place our results in the context of previously published international studies. We deliberately did not choose the format of a systematic literature review, with or without meta-analysis, so as not to restrict the inclusion criteria and thus provide an overview of the comprehensive number of studies on the most diverse aspects concerning this topic during this extraordinary period worldwide.

## Methods

### Patients admitted to the paediatric trauma centre of University Hospital RWTH Aachen

This part of the present study was performed according to the guidelines for reporting observational studies (STROBE) [32] and was approved and registered by the ethics committee of the University Hospital RWTH Aachen (project ID EK 22-320). The database of the Department of Orthopaedics, Trauma and Reconstructive Surgery, University Hospital RWTH Aachen/Germany (Level 1 trauma centre) was accessed. All patients younger than 18 years who were hospitalised with trauma diagnoses between January 1, 2019, and December 31, 2020, were included. Data from the pre-pandemic year January 1, 2019, to December 31, 2019, were compared to the pandemic year January 1, 2020, to December 31, 2020. In addition, the period of highly restrictive regulations (HRP), from the closure of kindergartens and schools until the reopening of both institutions, was evaluated separately (March 16, 2020, to June 7, 2020, versus the same period in 2019). The following endpoints were compared: length of hospitalisation, age of patients, place of lesion, type of lesion, activity during injury, and type of management. Observational treatments were carried out for injuries in which life-threatening conditions could occur after a symptom-free interval, i.e. traumatic brain injuries or blunt thoracic or abdominal injuries. Conservative treatment included injuries, mostly contusions and sprains, and more rarely fractures, that did not require surgical treatment, but which initially could not be treated at home due to severe pain and immobility.

The data were recorded in a database in Microsoft® Excel®. Statistical analyses were performed using the software IBM SPSS version 29. For the categorical variables, Pearson’s chi-squared test was used to determine whether there was a statistically significant difference between the expected frequencies and the observed frequencies of the contingency table. After checking for normality using the Shapiro–Wilk test, continuous data were analysed using the Mann–Whitney U test. Values of *p* < 0.05 were considered statistically significant.

### Literature review

On April 29, 2023, a PubMed search was performed with the search terms and Boolean operators listed in Table 1. From the resulting 177 records, those that contained information on the development and care of children’s injuries before, during, and sometimes after the COVID-19 pandemic were selected. Ninety-two records that matched this topic were selected for full-text screening. In studies that included both adults and children, only data from children were considered. On September 26, 2023, a final search with the same scheme was done, and six further reports were found.

**Table 1:** Literature search strategy

Search terms and Boolean operators	Corona OR COVID OR SARS-CoV-2	Paediatric OR Pediatric OR Child*	Fracture
Found records	364,965	3,558,746	
	Combined with AND 48,722		362,529
For title/abstract screening	Combined with AND 177		
For full-text screening	92 + 6		
Used for data description	79		

The records were reviewed according to the aspects author and publication year, study period, study institution/region, considered patients, and main findings regarding the following:

- Trauma-associated paediatric surgery
- Incidences/numbers of treated patients or injuries
- Special injury types, locations, or aetiologies
- Changes in the treatment process

## Results

### Patients admitted to the paediatric trauma centre of University Hospital RWTH Aachen

The data of 865 patients were collected: 445 from 2019 and 420 from 2020. Of these, 57 patients came to the trauma centre during the highly restricted period (HRP) in 2020 and 125 in the same period in 2019. We observed a 5.6% reduction in admissions over the entire 2020 and a 54.4% reduction in the HRP of 2020.

There was no significant difference in age between the two groups over the entire year or in looking only at the HRP. In the pandemic year 2020, the length of hospitalisation was longer compared to the pre-pandemic year 2019 (*p* = 0.045) (Table 2).

**Table 2:** Comparison of age and length of hospitalisation (significant differences in bold)

Endpoint	2019 (*n* = 445)	2020 (*n* = 420)		HRP 2019 (*n* = 125)	HRP 2020 (*n* = 57)	
Age (years ± SD)	8.6 ± 4.9	8.4 ± 5.0	*p* = 0.631	9.2 ± 4.5	7.9 ± 5.5	*p* = 0.112
Hospitalisation (days ± SD)	2.2 ± 2.7	2.4 ± 2.6	***p* = 0.045**	2.2 ± 2.3	2.7 ± 3.9	*p* = 0.175

Regarding the place of the lesions, there were no differences either for the entire year or for the HRP (Table 3). There were also no differences in the type of lesions except during the pandemic year when significantly more wounds requiring surgical therapy were the reason for hospitalisation (Table 4).

**Table 3:** Comparison of groups regarding the place of lesion

Place of lesion	2019	2020	*p*	HRP 2019	HRP 2020	*p*
Head	169	176	0.238	37	19	0.613
Clavicle	11	11	0.891	2	3	0.161
Shoulder	2	1	0.597	2	0	0.337
Upper arm	6	4	0.586	0	0	
Elbow	31	30	0.919	10	6	0.577
Forearm	76	72	0.980	25	14	0.487
Hand	1	1	0.967	0	0	
Thorax	4	3	0.762	0	0	
Abdomen	11	10	0.931	3	1	0.783
Spine	21	17	0.630	9	1	0.135
Pelvis	7	2	0.112	2	1	0.940
Hip	2	1	0.597	0	0	
Upper thigh	15	13	0.819	7	2	0.546
Knee	12	9	0.597	3	2	0.671
Patella	2	1	0.597	0	0	
Lower thigh	18	16	0.859	4	3	0.502
Foot	7	11	0.281	4	1	0.580
Multiple	50	42	0.556	17	4	0.197
Total	**445**	**420**		**125**	**57**

**Table 4:** Comparison of groups regarding type of lesion (significant differences in bold)

Type of lesion	2019	2020	*p*	HRP 2019	HRP 2020	*p*
Blunt trauma of body cavities	8	12	0.300	2	1	0.940
Cartilage damage	4	0	0.051	0	0	
Commotio cerebri	123	141	0.058	32	13	0.685
Contusion of the extremities	35	28	0.498	7	6	0.231
Joints sprain	8	3	0.155	4	0	0.172
Electrical accident	0	1	0.303	0	0	
Exclusion of consequences of injury	1	2	0.530	0	0	
Foreign body	1	1	0.967	0	0	
Fracture	169	141	0.177	50	27	0.351
Trauma-related infection	5	3	0.530	1	1	0.567
Injury of abdominal organ	2	0	0.169	1	0	0.498
Ligament and/or meniscus tear	4	3	0.762	2	0	0.337
Luxation of joints	4	2	0.454	2	1	0.940
Multiple injuries	76	67	0.656	22	6	0.220
Polytrauma	3	7	0.172	1	2	0.183
Traumatic amputation	1	0	0.331	1	0	0.498
Wounds requiring surgical therapy	1	9	**0.008**	0	0	0
Total	**445**	**420**		**125**	**57**	

In 2020, there was a significant reduction in admissions of patients with lesions that were treated conservatively (*p* = 0.005) and an increase of patients who were hospitalised for observation (*p* = 0.046). However, there was no significant change in surgical cases between the two entire years. In the HRP, no significant changes were observed in any of these aspects (Table 5). There was no significant difference in whether the injury took place during free time or school/kindergarten time over the entire years 2019 and 2020. However, this was significantly the case during the HRP (*p* < 0.001) (Table 6).

**Table 5:** Comparison of management (significant differences in bold)

Management	2019	2020	*p*	HRP 2019	HRP 2020	*p*
Conservative	63	34	**0.005**	15	5	0.518
Observation	204	221	**0.046**	57	21	0.268
Surgical	178	165	0.830	53	31	0.132
Total	**445**	**420**		**125**	**57**

**Table 6:** Comparison of activities during injury (significant differences in bold)

Activity	2019	2020	*p*	HRP 2019	HRP 2020	*p*
Free time	283	285	*p* = 0.187	66	52	***p* < 0.001**
School/kindergarten	162	135		59	5	
Total	**445**	**420**		**125**	**57**	

### Reported results in literature

**Fig. 1:**
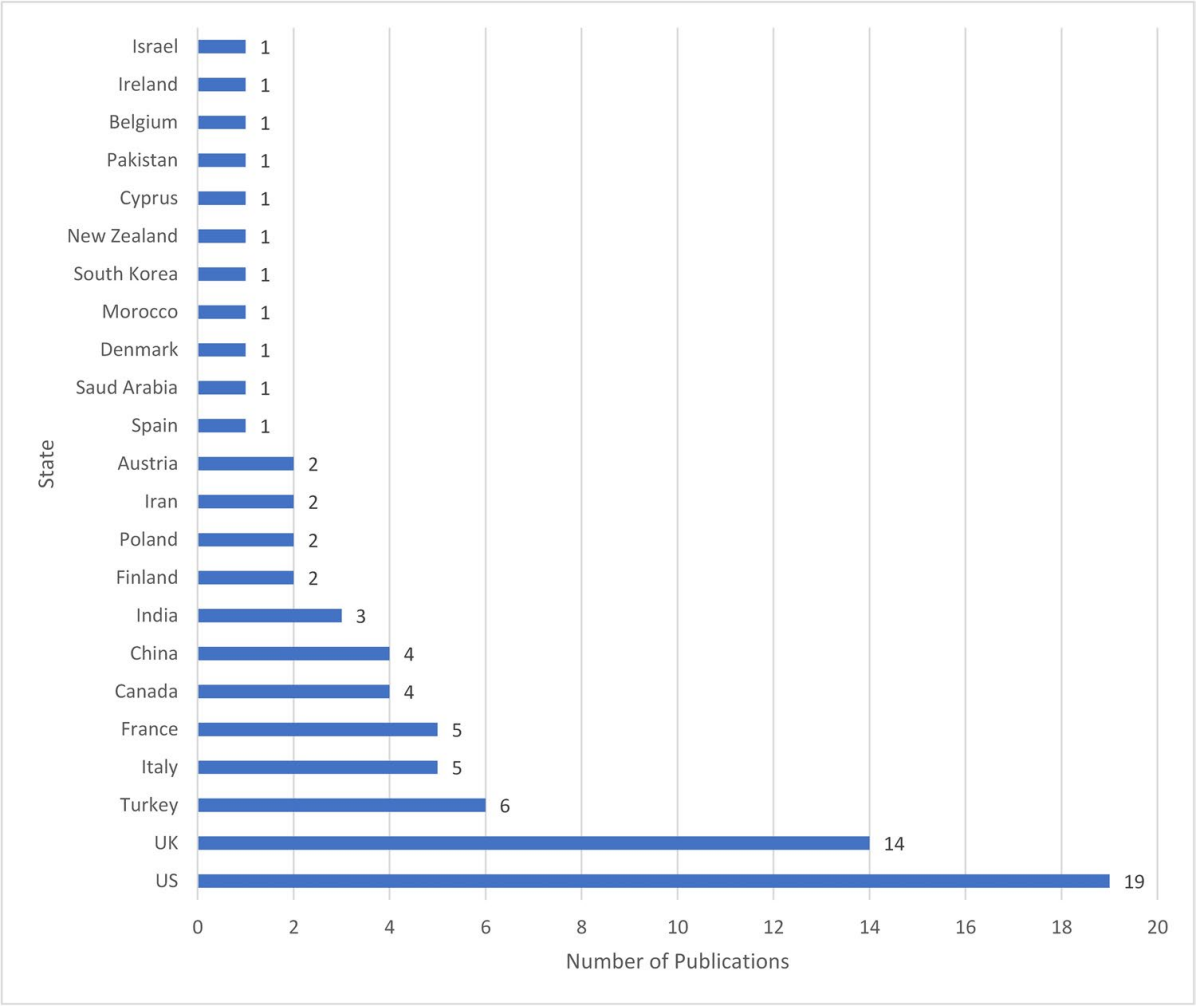
Origin of publications per state

Using the above-mentioned search criteria, 79 publications dealing with paediatric patients presenting to trauma departments during the COVID-19 pandemic were identified. These studies are listed in Table 7 to Table 10 of the supplement, with a presentation of the main findings and the investigation period. Twenty studies commented on surgical aspects (Table 7), 28 studies on general treatment numbers (Table 8), 24 studies on specific injury regions or types (Table 9), and 7 studies on changes in the treatment process (Table 10). Most publications came from Europe, North America, and Asia (Fig. 1).

#### Changes in surgical management and absolute numbers of treated patients

In accordance with our results, most studies describe a decrease in the absolute numbers of treated patients or injuries during the lockdown in 2020 (Supplement, Table 8) and no significant change in the proportion of surgically managed patients (Supplement, Table 7) [1–4, 13, 14, 33–37]. However, in a few studies, divergent changes in surgical management, with either a preference for non-operative management of lower leg fractures [38] or a significantly elevated frequency of operative treatment during the lockdown [12, 39, 40], are described. Simon et al. reported altered management, without complications: elbow pins left protruding were removed in consultation rather than in the outpatient surgery room, as was done previously. Under general anaesthesia, only overriding frontally misaligned fractures of the distal quarter of the two forearm bones were reduced [27]. We did not change our surgical procedures for our patients in 2020.

#### Injury types, locations, and aetiologies (Supplement, Table 9)

##### Domestic injuries

Our results show a significant decrease in injuries sustained during the HRP in public facilities. In the literature, during the pandemic, the majority of studies reported an increase in the proportion of injuries occurring in the domestic environment [4, 8, 23, 26, 41–44], whereas the incidence of outdoor injuries acquired in public areas or in sports clubs significantly decreased [8, 23, 26, 42, 44, 45].

##### Sports-related injuries

In line with the above findings, sports-related injuries were less likely to occur during the lockdown [11, 15, 38, 41, 45–47]. It might be suggested that the nationwide lockdown and the cancellation of sports and other hobbies markedly decreased injuries among children aged 13–17 years, while the decrease was lower among younger children, as the cancellation of sports and hobbies did not affect patients in this age group as much as it did adolescents [13]. However, many studies have found a significantly younger age of patients requiring admission or surgery during the lockdown [2, 3, 5, 7–9, 12, 20, 25, 26, 29, 31, 34, 38, 39, 42, 46, 48–50]. Although not significantly, the mean age of our patients was also lower during the pandemic.

##### Fracture and injury patterns

In accordance with our results, in a study from Colorado, USA, there was no significant shift in the frequency of upper extremity fractures, lower extremity fractures, and axial/pelvic fractures [26]. However, some studies have shown a shift towards an increased occurrence of certain types of fractures in specific body regions [12, 14, 18, 38, 39] or a decrease in them [3, 12]. Other studies have reported that wound therapy was more common during the pandemic, as our study did [3, 8, 16, 20].

##### Mild traumatic brain injuries

The increase in mild traumatic brain injuries (commotio cerebri) only just missed the significance level in our patients. However, these injuries account for most hospitalised childhood injuries that present in our clinic for observation, and that proportion increased significantly in our patient population in 2020. Payr et al. also found a significant increase in mild traumatic brain injuries during the lockdown period [20].

##### Child abuse and neglect

Regarding non-accidental trauma (NAT), we have not yet carried out any analysis of our patients. However, the literature indicates that falls from windows were significantly elevated during the California-wide shelter-in-place mandate [51]. Some studies have reported significantly higher cases of child abuse and neglect during lockdown [9, 52]. Furthermore, the severity of abusive head trauma (AHT) worsened in 2021 in terms of mortality [53]. In contrast, McCauley et al. analysed American data and did not find significant changes in the frequency of NAT or the risk of fracture due to NAT [54]. Furthermore, no difference between positive/negative radiographic skeletal survey (SkS) rates was found [55, 56].

##### Vehicle accidents

We did not evaluate the detailed causes of these injuries in our patients, but there are reports that traffic restrictions resulted in a reduction in fractures associated with traffic accidents [48]. However, due to reduced public transport, children and young people were likely to use bicycles more often, so bicycle injuries were significantly higher during the pandemic [57–60].

#### Management aspects (Supplement, Table 10)

In our study, we did not examine possible changes in the treatment processes of injured children and adolescents. However, the literature contains some interesting findings in this regard, such as the higher effectiveness of conservative and surgical treatment processes.

In this context, changes in the management of conservatively treated fractures showed significant potential for cost savings through reduced numbers of clinic appointments and cast removals, without increased complication rates [61]. In terms of surgical management, the average waiting times for operations dropped significantly, with patients waiting 80% less time from the date of their injury [38]. Sabaghzadeh et al. also reported lower times for the transfer of patients to the operating room [43].

Memeo et al.’s study in Milan, Italy, found a reduction of paediatric visits to the ER, with an increased ‘positive’ fracture and surgery rate due to the government’s reorganisation of patient flow to hospitals, which involved the creation of more effective outpatient services, with only severe cases presenting to the ER [17]. Accordingly, Sugand et al. reported a greater use of outpatient telemedicine in the COVID-19 period, with more virtual fracture clinic use and, subsequently, fewer patients seen for consultation and followed up face to face [28]. These changes also explain the reduction in the proportion of negative studies (with no evidence of bone fractures in radiological diagnostics) by about one-fifth [62].

Further measures to reduce in-hospital care during the pandemic included the development of guidelines for the more efficient treatment of distal forearm fractures. There was high compliance with these guidelines after the pandemic [63]. Moreover, the increased use of easily accessible media for patient education has been shown to be effective regarding paediatric humeral supracondylar fractures during the pandemic [64].

#### Postpandemic period and further aspects in the literature

The years after 2020 were not considered in our study, but the literature shows interesting results for this period. For example, a trend towards decreased injuries persisted for 12 months after the peak of the pandemic [5], and the number of paediatric orthopaedic surgery visits did not return to prepandemic levels in 2021 [6]. In contrast, a study by Kalem et al. [35] found that after the first 3 months following the lockdown, the prevalence of outdoor traumas, high-energy traumas, the rate of patients treated with surgery, and the rate of admission to the emergency department were significantly higher than in the previous year. The authors assumed that children’s lower extremity muscle strength and neuromuscular control decreased after staying at home for a prolonged period. In addition, in investigating the reasons for a higher fracture rate, Lee et al. [65] found a higher prevalence of vitamin D deficiency in healthy Korean children with fractures during the pandemic.

## Discussion

The data collected from hospitalised children in the paediatric trauma centre of our university hospital in North Rhine-Westphalia/Germany show that the COVID-19 lockdown in 2020 resulted in a 5.6% drop in treatments compared to 2019, without a significant change in the average age of patients, locations, and most types of injuries. However, the year-on-year comparison revealed a longer hospitalisation time, an increased proportion of wounds requiring surgical therapy, a lower proportion of conservative treatments, and an increased proportion of injuries that required observation. The proportion of surgical treatments did not change. Looking at the highly restrictive period (HRP) of the 12-week lockdown, the absolute number of cases fell by 54.4% compared to the respective period in 2019. Again, there were no differences in average patient age, hospitalisation length, location, type of lesion, or management. As expected, there was a highly significant difference in terms of whether the injuries occurred in school or kindergarten or outside these facilities.

Decreased absolute numbers of admitted and hospitalised children in paediatric traumatology departments during the pandemic are in accordance with the published literature (Supplement, Table 7 to Table 10), although in our population, this was only very clear in the HRP. The discussed and obvious reasons for this are the stay-at-home order and the associated lower exposure to injury-prone activities and settings that require a hospital visit (e.g. playgrounds, sports clubs, and traffic) [24].

The longer hospitalisation time in 2020 in our patient clientele can most likely be explained by a lack of resources among operating room staff due to high levels of sick leave over the entire year, meaning that patients waited longer for their operations. The slightly increased number of patients who had injuries that necessitated observation probably also resulted in longer hospital stays. Children who undergo surgery are usually discharged the day after the operation, while observations are usually scheduled for 48 h. The injuries observed in our department were mostly mild traumatic brain injuries, which increased in 2020, although the significance threshold was narrowly missed.

The increase in mild traumatic brain injuries is also reflected in the study by Payr et al. in Vienna, also a Level I trauma centre in Europe [20]. The authors explained that this change could be explained by the increased number of parents working from home who had to look after their children at the same time and who may not have been able to give them their full attention in order to avoid injuries. The significantly increased proportion of treated wounds in 2020 in our population may also be due to this fact, which Payr et al. and Mason et al. also observed [16, 20]. The question of why this is only statistically noticeable over the entire year and not just in the HRP probably has statistical reasons due to the lower number of patients during the HRP. However, working from home and video conferencing options were not yet established in Germany at the start of the lockdown, and possibilities to work from home in the official sense were only implemented later in the year. However, this hypothesis needs to be substantiated with a targeted study on the risk of injury among children who are cared for by people working from home.

Similar to our study, an unchanged proportion of fractures was also observed in the study from Vienna mentioned above [20], an excellent comparable study in which the authors compared the entire year of 2020 with previous years’ injuries at a Level 1 trauma centre. In our literature review, we included publications that dealt with childhood injuries during the pandemic, most of them from the USA and the UK. The reports regarding the decrease, or sometimes increase, in treated patients, injuries, rate of fractures, mild injuries, and number of surgical cases vary between the studies. The most likely reason for this is the different structure of the clinics or databases, their catchment areas, their capabilities or sizes, the period of study, and the differences in healthcare and insurance, as well as the statistical methods used.

Regarding childhood fractures, in 2022, Oh et al. [19] published a review of the epidemiology of paediatric fractures before and during the pandemic, which considered studies from all over the world [2–4, 25, 38, 39, 41, 48]. They concluded that fractures in adolescents due to sports activities decreased relative to those in younger children with more low-energy trauma. This shift is seen as the main reason for the younger mean age of injured children during the pandemic. In our patients, including not only fractures, the mean age in 2020 was also younger, but the significance level was not reached.

A further aspect is the significantly reduced proportion of conservative treatments in 2020 in our patients. Similar to other studies, it is probable that only children with major trauma were brought to the hospital by their parents to avoid exposure to the virus, whereas children with contusions and sprains were treated conservatively at home [7]. It is worth considering whether this might prove that inpatient resources could be conserved in a more restrictive inpatient admission regime. This aspect should be used as an opportunity to review the criteria for the hospitalisation of injured children as part of the restructuring of the healthcare system. Therefore, further studies are desirable. In this context, also safe outpatient treatment options have to be ensured. However, the concern that conservative treatment decisions may have been favoured due to the scarcity of resources in the operating room has not been confirmed. The idea that compromises were made in surgical decisions due to reduced resources is not supported by the published literature and our results. Only in the study of Darling et al. was a preference for non-operative management in lower limb fractures reported [38]. We were unable to find many studies that explicitly reported that patients who would have had surgery before the pandemic were treated conservatively during it. The changes in the number of surgical cases were more likely due to changes in injury patterns and absolute patient numbers.

The studies that examined the handling of patient volumes during the pandemic in more detail revealed interesting results in relation to more efficient procedures, particularly in the context of follow-up treatment and the use of X-rays without worsened outcomes [10, 28, 61–63]. Reduced mobility and more domestic activities are also sustainable factors to consider in bone health density in children during possible future lockdowns or in times of suspected reduced activity in children [35, 65, 66].

Our literature search revealed that this study is the only one to date to report data from a university paediatric trauma centre in Germany. The Robert Koch Institute (RKI) is the German government’s central scientific institution in the field of biomedicine. It is one of the most important bodies for safeguarding public health in Germany. On May 10, 2023, this institution published a 66-page report entitled ‘Monitoring of Children’s Health in (and after) COVID-19 Pandemic’, which described the situation that detailed data from Germany are missing [67]. We aim to contribute to closing this gap through our data.

### Limitations

In our patient clientele, we compared only the entire years of 2019 and 2020, as well as the highly restricted period in 2020, with the respective period in 2019. To exclude annual fluctuations due to other causes, a comparison with the years before and after the pandemic would be interesting.

We examined only injuries that resulted in hospitalisation. This meant that the more serious injuries could be recorded reliably, the evaluation of a defined organizational structure was guaranteed, and, with manageable case numbers, a more detailed evaluation could be carried out on the basis of reviewed patient records, not just on the basis of DRGs. However, changes in injuries treated on an outpatient basis are also necessary to illuminate the findings.

Our data came from a Level 1 trauma centre that did not turn away injured children during the pandemic and had few restrictions or triages. This means that our data cannot be transferred to smaller hospitals. The investigation of the transfer behaviour from other hospitals to ours and the areas where children and families came from during this time would be interesting for future optimisation efforts in the healthcare system.

We have not performed a comprehensive, detailed analysis of the causes of injury, which may be desirable for future preventive considerations in a variety of areas. However, these data were not consistently stored in the medical records. It would be particularly interesting to look at child abuse and non-accidental trauma during this period.

In our search strategy, the use of the term ‘fracture’ probably excluded some studies only dealing with soft-tissue injuries, mild brain injuries, or others. However, this made comparability with our patient clientele more feasible.

## Conclusions

When comparing childhood injuries in the entire years 2019 and 2020, as well as patient numbers, there were slight differences in our patients, although there is a downward trend, as can be seen in most published studies on this topic.

The indications for surgical treatment of childhood injuries are so strict and clear that no or only minor compromises towards conservative approaches were made, even with limited resources.

Further studies should examine whether childhood injuries sustained in the domestic environment and requiring hospitalisation were caused by excessive demands on parents or carers and simultaneous workloads especially when opportunities to work from home are increasingly being established in the future.

Reduced inpatient conservative treatment during the pandemic implies that hospital resources may have been overused previously. This means that as the healthcare system is restructured, ways could be found to ensure efficient outpatient treatment without compromising safety.

With strict inclusion and exclusion criteria, the existing literature has much potential to answer detailed questions regarding childhood injuries during a pandemic, as well as to highlight possible changes in treatment processes. These data should also be used to reduce the burden on emergency rooms and clinics when treating injured children, without reducing their safety.

### Supplementary information

Below is the link to the electronic supplementary material.

Supplementary file1 (PDF 174 KB)

Supplementary file2 (PDF 156 KB)

Supplementary file3 (PDF 158 KB)

Supplementary file4 (PDF 126 KB)

## Data Availability

A de-identifed dataset associated with the paper is available upon reasonable request.

## Abbreviations

*vs*: Versus
*ED*: Emergency department
*ER*: Emergency room
*SCH*: Supracondylar humerus
*SkS*: Radiographic skeletal survey
*BOAST*: British Orthopaedic Association Standards for Trauma and Orthopedics
*HRP*: Highly restricted period
*SD*: Standard deviation
*PCA*: Physical child abuse
*ISS*: Injury Severity Score
