# Supplementary material for: Influence of COVID-19 pandemic on hospitalisations at a paediatric traumatology department during 2020: a single-centre observational study and comprehensive literature review
Source: Eur J Trauma Emerg Surg. 2024 Jan 30;50(2):591–601. doi: 10.1007/s00068-024-02453-7 (PMC11035450; doi:10.1007/s00068-024-02453-7)
Supplement: Supplementary file 1 — Supplementary file1 (PDF 174 KB) [file 68_2024_2453_MOESM1_ESM.pdf]

Table 7 Findings of studies regarding proportion of trauma-associated paediatric surgeries

Influence of COVID-19 pandemic in hospitalisations at a paediatric traumatology department during 2020: A single-centre observational study and comprehensive literature review

European Journal of Trauma and Emergency Surgery

Heide Delbrück*, Ellen Lambertz, Filippo Migliorini, Nina Berger, Frank Hildebrand

*Correspondence: hdelbrueck@ukaachen.de; ORCID 0000-0002-1676-4115

Author	Region	Considered patients	Periods	Main findings regarding pandemic period
Abunayan et al., 2022 [1]	Riydh, Saud Arabia, tertiary hospital	Adult and paediatric orthopaedic procedures	2020/01/01–2020/06/30 vs. same period in 2019	No significant difference in paediatric emergency surgery and paediatric elective surgery
Baxter et al., 2020 [2]	Sheffield Children's NHS Foundation Trust, Sheffield, UK	Paediatric admissions	2020/03/24–2020/05/10 vs. same period in 2019	Operative rate not significantly changed: 77.6% (n = 153) vs. 72% (n = 95)
Bolzinger et al., 2023 [3]	University Hospital of Toulouse, France	Trauma cases to be managed by the paediatric orthopaedic and traumatology team	2020/03/17–2020/05/10 vs. same period in 2017, 2018 and 2019	Surgically managed patients during lockdown 86% of those in previous years (not significant)

Bram et al., 2020 [4]	Division of Orthopedics, Children's Hospital of Philadelphia; Perelman School of Medicine, University of Pennsylvania, Philadelphia, single Level I paediatric trauma hospital	Acute fractures	2020/03/15–2020/04/15 vs. same period in 2018 and 2019	Proportion of patients requiring surgery was similar; decrease in the number of fractures requiring surgery
Carkci et al., 2021 [68]	Orthopedics and Traumatology, Istanbul Training and Research Hospital; Dr. Burhan Nalbantoglu State Hospital, Nicosia, CYP; Dr. Akçiçek State Hospital, Kyrenia, CYP	Orthopaedic trauma (adult and paediatric) presented in ED	2020/04/12–2020/05/12 vs. same period in 2019	Significant decrease in the number of fractures requiring surgery
Darling et al., 2022 [38]	Wythenshawe Hospital, Manchester Foundation Trust, Manchester, UK	67 paediatric patients with lower limb fractures	2019/08/28–2021/04/01	Drop in the number of referrals and a preference for non-operative management ($p = 0.0016$)
Elbardesy et al., 2021 [33]	Trauma and Orthopedic Department at Cork University Hospital; South Infirmary Victoria University Hospital, Level 1 Trauma Centre in the Republic of Ireland	Patients presented to the emergency department and required surgical intervention by the trauma and orthopaedic department's team	2020/03/01–2020/04/15 vs. same period in 2019	Number of operations performed on children fell from 62 to 37, denoting a 40.32% reduction ($p = 0.15$).
Gokhale et al. 2021 [8]	Paediatric orthopaedic unit at a tertiary referral centre in Wales, UK	Paediatric (0–16 years) patients managed by the paediatric orthopaedic unit	2020/03–2020/07 vs. same period in 2019 and 2021	During lockdown, there was a 48% reduction in the number of patients who required intervention in the operating theatre.

Hampton et al., 2020 [34]	Paediatric major trauma centre, UK	Patients admitted by orthopaedic team, trauma admissions and surgical cases	2020/03/10–2020/03/23, 2020/03/24–2020/04/07 vs. same period in 2019	No significant changes in injury types; no difference between percentage of cases admitted that required surgical intervention between the time periods
Kalem et al., 2021 [69]	Department of Orthopedics and Traumatology, İbn'i Sina Training and Research Hospital, Ankara University, School of Medicine, Ankara, Turkey	Fractures or soft-tissue trauma; 3 subgroups according to age of patients (≤ 20 years, 21–64 years and ≥ 65 years)	2020/03/21–2020/06/01 vs. same period in 2019	Number of admissions decreased significantly by 50.9%; rate of high-impact traumas decreased; surgical treatment was not significantly different.
Karia et al., 2020 [70]	Royal Berkshire Hospital, Reading, UK	Patients admitted by the orthopaedic team or sent home following review	2020/04/01–2020/04/30 vs. same period in 2019	Paediatric admissions decreased by 72%; paediatric admissions/surgery in 2019 were 32/18 vs. 9/6 in 2020.
Köksal et al., 2022 [12]	Level III trauma centre in Istanbul	Orthopaedic paediatric trauma admissions	2020/04/03–2020/05/31 vs. same period in 2019	Frequency of operative treatments was significantly higher in lockdown group ($p < 0.001$).
Kuorikoski et al., 2021 [13]	Three large Finnish hospitals (Tampere University Hospital, Mikkeli Central Hospital and Central Finland Hospital)	ED visits due to paediatric trauma and paediatric trauma surgeries	2020 vs. 2017–2019	Paediatric trauma visits decreased; incidence of head injuries and sprains decreased in the groups aged 4–12 year and 13–17, while the incidence of fractures decreased only in the 13–17 age group; incidence of paediatric trauma surgery remained stable.

Lapsa et al., 2022 [14]	Pediatric Emergency Medicine, Johns Hopkins University, Baltimore, US (large urban tertiary care paediatric centre)	Paediatric emergency department (PED) patients evaluated for fracture	2020/03/30–2020/09/05 vs. same period in 2019 and 2018	40% decrease in the number of patients presenting to the PED with fractures compared with 2019 and a 28% decrease from 2018; no statistical difference in the proportion of fractures requiring closed reduction under sedation or operative repair on initial presentation
Mason et al., 2022 [16]	Starship Children's Health, New Zealand	Orthopaedic presentations, seen by either the Children's Emergency Department (CED) or the orthopaedic service	2020/03/26–2020/05/29 vs. same period in 2019	Overall reduction of 37%; 55% reduction in musculoskeletal infections; 40% reduction in total fractures; 27% reduction in soft tissue injuries; similar proportions of patients were admitted for operating room procedures (15%) or had fractures reduced under sedation in ED (17%); increased numbers of soft-tissue injuries managed through ED under procedural sedation
Murphy et al., 2020 [71]	District General Hospital (Gloucester Royal Hospital), UK	Referrals to the on-call orthopaedic service	2020/03/09–2020/04/26 vs. same period in 2017–2019	Significant reductions in the rates of simple fractures and the most common fracture surgeries in children
Olech et al., 2021 [36]	Orthopedic Surgery Department, Legnica, Poland; Department of Orthopaedic and Trauma Surgery, Institute of Medical Sciences, University of Opole, Poland	Treatment of distal radius fractures	2020/03/15–2020/10/15 vs. same period in 2019	No significant change in number of hospitalised children or in conservative and surgically treated children; significant reduction in average length of hospitalisation in the case of surgical treatment of children

Simon et al., 2023 [27]	Hôpital Universitaire Robert Debré, Paris, France	Children < 18 years undergoing paediatric orthopaedic emergency surgery	2020/03/17–2020/05/11 vs. same period in 2017–2019	Turnover fell by a mean 33.5%, without a change in indications; postoperative management was modulated during lockdown in 34% of cases: elbow pins were left protruding, under general anaesthesia only, overriding frontally misaligned fractures of the distal quarter of the two forearm bones were reduced.
Turgut et al., 2020 [39]	Department of Orthopedics and Traumatology, Tepecik Training and Research Hospital, İzmir, Turkey	Admissions to the hospital with a new fracture, two groups: ≤ 16 years (group 1) and >16 years (group 2)	2020/03/16–2020/05/22 vs. same period in 2018 and 2019	Frequency of fractures decreased by approximately one-third; proportional increase in femoral and tibial shaft fractures in group 1; rates of operations were 6.8%, 7.7% and 14.6% in the 2018, 2019 and 2020 periods, respectively ($p < 0.001$).
Yavuz et al., 2021 [37]	Department of Orthopedics and Traumatology, Health Science University, Ankara Bilkent City Hospital, Ankara, Turkey	Patients who were operated on (two groups: aged > 16 and < 16)	2020/04/01–2020/05/31 vs. 2020/09/01–2020/10/31 vs. 2019/09/01–2019/10/31	No statistically significant difference regarding the distribution of the number of operated paediatric fractures in the three time periods
