# Supplementary material for: Influence of COVID-19 pandemic on hospitalisations at a paediatric traumatology department during 2020: a single-centre observational study and comprehensive literature review
Source: Eur J Trauma Emerg Surg. 2024 Jan 30;50(2):591–601. doi: 10.1007/s00068-024-02453-7 (PMC11035450; doi:10.1007/s00068-024-02453-7)
Supplement: Supplementary file 2 — Supplementary file2 (PDF 156 KB) [file 68_2024_2453_MOESM2_ESM.pdf]

Table 8 Findings of studies regarding incidences, number of treated patients and injuries

Influence of COVID-19 pandemic in hospitalisations at a paediatric traumatology department during 2020: A single-centre observational study and comprehensive literature review

European Journal of Trauma and Emergency Surgery

Heide Delbrück*, Ellen Lambertz, Filippo Migliorini, Nina Berger, Frank Hildebrand

*Correspondence: hdelbrueck@ukaachen.de; ORCID 0000-0002-1676-4115

Author	Region	Considered patients	Periods	Main findings regarding pandemic period
Butler et al., 2023 [5]	Department of Orthopedic Surgery, Icahn School of Medicine at Mount Sinai, New York City, US	Paediatric patients with trauma in ED or outpatient department	2018/01/01–2021/11/30	Decreased trauma and fracture rates persisted for the post-peak pandemic cohort.
Choi et al., 2021 [72]	Six hospitals in large cities in South Korea	Patients < 18 years who visited paediatric emergency departments	2017/01/01–2020/11/08	Absolute numbers decreased by 43.6%; the proportion of paediatric patients with injuries increased by 8.6%.
Culbert et al., 2023 [6]	Division of Pediatric Orthopedic Surgery, University Hospitals Rainbow Babies and Children's Hospital, Cleveland, OH, US; single-centre, multisite paediatric Level 1 trauma centre	All patients seen in the Division of Pediatric Orthopedics (outpatients and emergency room)	May to August 2019, 2020 and 2021	Visits decreased by 22.2% during the pandemic; 11.6% lower after vaccine availability compared with prepandemic; total fractures were 13.4% lower than prepandemic period; elective visits were 13% lower than postpandemic period.
Dhillon et al., 2020 [73]	Tertiary care referral centre in North India	Orthopaedic emergency with fresh injuries, or those that were referred for tertiary level management	Phase 1: 2020/03/25–2020/05/03<br>Phase 2: 2020/05/04–2020/05/31<br>vs. same periods in 2019	In 2020, nine paediatric patients were admitted during phase 1 (0.22 admissions per day); during phase 2, this increased to 17 (0.6 admissions per day); in 2019, there were 0.62 and 0.82 admissions per day, respectively.

Dibello et al., 2021 [7]	Second-level paediatric trauma centre, Italy	Children admitted to paediatric orthopaedic unit with a diagnosis of fracture or trauma, including sprains and contusions	2020/03/11–2020/04/11 vs. same period in 2018 and 2019	In 2020, 69 patients were admitted (significant decrease of about 75%); fracture rate significantly increased in 2020.
Greenhalgh et al., 2021 [74]	Lancashire Teaching Hospitals, major trauma centre in UK	Patients referred to the orthopaedic team (adults and children)	2020/03/16–2020/04/22 vs. same period in 2019	Decrease of 53.6% in paediatric referrals (56 in 2019, 26 in 2020)
Hashimi et al., 2020 [75]	Orthopedic Surgery, Aga Khan University Hospital, Karachi, Pakistan	Admitted patients (adults and children)	2020/02/01–2020/04/30, divided into pre-COVID and COVID periods (6 weeks each)	No significant change in numbers of supracondylar humerus (4 vs. 5) and distal femur fractures (1 vs. 2)
Hennocq et al., 2022 [9]	Greater Paris region, 20 hospitals	All paediatric admissions at emergency and critical care departments; injuries and child abuse and neglect	2020/03/17–2020/05/11 vs. same period in 2019	During lockdown, 12,942 patients (including 476 injuries, 3.7%) were admitted vs. 42,113 patients (including 923 injuries, 2.2%) during the control period ($p < 0.001$); child abuse and neglect represented 18 cases during the 2020 lockdown (0.139%) vs. 24 cases in 2019 (0.057%) ($p = 0.005$).
Hernigou et al., 2020 [40]	Two sites of hospital CH EpiCURA: Baudour and Hornu, Belgium	Each patient had an admission diagnosis of fracture, trauma, sprain, dislocation or wound	2020/03/01–2020/04/15 vs. same period in 2018	Number of surgically treated children significantly higher (8 fractures in 2020 and 3 in 2018)
Keays et al., 2020 [11]	Montreal Children's Hospital, a provincially designated pediatric trauma centre, Canada	Injury-related emergency department visits	2020/03/16–2020/05/15 vs. same period 1993–2019	ED visits by children aged 0 to 17 years decreased by 72% compared to the average of the previous 5 years; more children with less urgent injuries
Li et al., 2021 [48]	Children's Hospital of Chongqing Medical University, Southwest China	Admitted children under age 16 with a traumatic injury	2020/01/24–2020/03/10 vs. same period in 2017, 2018 and 2019	Fracture rate among children with trauma was 55.01% in period 2017–2019, significantly higher than 46.12% in 2020; fracture rate in males was higher in 2017–2019 than in 2020.

Lv et al., 2022 [76]	18 hospitals from China	New-onset fractures, 6,196 children	2020/01/10–2020/07/10	With the increase in newly confirmed COVID-19 cases, the fracture risk decreased in children.
Markiewitz et al., 2022 [15]	National Electronic Injury Surveillance System (NEISS) database and the American Community Survey (ACS), US	Paediatric fractures, (2,959,421 ± 372,337 fractures)	Between 2016 and 2020	Average annual fracture incidence of 8.47 per 1,000 youth years from 2016 to 2019 and 6.55 in 2020, with a 27% decrease during the COVID-19 pandemic.
Moore et al., 2022 [57]	Mercy Health St. Vincent Medical Center, Toledo, US	Trauma Registry, paediatric patients (< 15 years)	2020/03/01–2021/02/28 vs. same period one year prior	Paediatric encounters increased by 6.4% during the COVID-19 pandemic.
Nabian et al., 2020 [18]	Taleghani tertiary trauma centre, Iran	Referrals to tertiary trauma centre	2020/03/01–2020/04/15 vs. same period in 2019 and 2018	52% decline in fractures; fractures of humeral supracondylar region, radioulnar shaft, distal forearm and proximal tibia showed a significant increase.
Pepper et al., 2021 [21]	Paediatric emergency department (PED) at Robert Wood Johnson University Hospital, New Jersey, US	Retrospective analysis of medical records of patients visiting the PED	2020/03–2020/07 vs. same period in 2018 and 2019	49% reduction in admissions during census nadir; sharp decline in numbers of fractures managed during the COVID-19 surge in April, after which there was a gradual month-to-month increase, reaching pre-COVID-19 experience in July.
Qian et al., 2021 [77]	Orthopaedic emergency department of National Children's Medical Center Shanghai, China	Orthopaedic emergency patients (< 18 years)	From 1 January to 30 June 2018, 2019 and 2020, three response levels	First-level response (2020/01/24–2020/03/22) number of fractures, open injuries, radial head subluxation and surgery were significantly reduced, and the severity of patients with surgery was also significantly reduced.
Raitio et al., [22]	Five tertiary centres for paediatric surgery and orthopaedics in Finland	Fracture-related paediatric (> age 16) emergency operations	2020/03/01–2020/05/31 vs. same period in 2017–2019	Frequency of all fracture operations reduced most significantly (31%) during March and April 2020, especially in lower limb fractures.

Rasmussen et al., 2022 [23]	Five regional hospitals and two university hospitals in the northern and middle parts of Denmark	All patients aged 0–15 admitted to emergency departments with paediatric musculoskeletal injuries	2020/03/16–2020/04/21 vs. same period in 2019	Decrease of 51%; the primary decrease in incidence between the pandemic and pre-pandemic cohorts was observed in adolescents.
Rougereau et al., 2021 [24]	Paediatric teaching hospital, Armand-Trousseau Hospital, Sorbonne University, Paris, France	Paediatric emergency department visits and hospitalisations for traumatic injuries	2020/02/16–2020/05/11 vs. same period in 2018 and 2019	Significant decrease in recorded visits, average number of visits per day and hospitalisations per day; number of visits per day not significantly lower for burns
Ruzzini et al., 2021 [25]	Pediatric Emergency Department (PED) of the Bambino Gesù Children's Hospital, Rome (Palidoro), Italy	Admission diagnosis for fracture, trauma, sprain and dislocation	2020/03/10–2020/05/04 vs. same period in 2019	Reduction of total traumas and fractures (81%); increase of rate of fractures and decrease of minor traumas
Salom et al., 2022 [50]	Three tertiary hospitals in Spain	Paediatric trauma emergencies	2020/03/15–2020/06/21 vs. same period in 2019	In the Madrid hospital, paediatric emergency care decreased by 83.5%, in the Valencia hospital by 75%, in the Palma de Mallorca hospital by 65.9%; proportion of moderate and severe conditions was higher.
Sephton et al., 2021 [45]	Five large NHS Trusts from the North West London Trauma Network, UK	Emergency orthopaedic referrals and procedures performed; also adults	2020/03/23–2020/06/14 vs. same period in 2019	Pushbike/scooter-related accidents proportionally increased significantly from 4.7% of injuries at baseline to 10.7% of injuries during lockdown; proportion of sports accidents and atraumatic injuries were significantly lower during the lockdown.
Shaw et al., 2022 [26]	Level I paediatric trauma centre in Colorado, US	Referred patients for definitive management of a fracture	2020/03/26–2020/05/31 vs. same period in 2018 and 2019	Number of fractures decreased by 26% resp. 23%; larger proportion of patients experienced at least a 5-day delay in definitive treatment; more fractures occurred at home; non-significant increase in fractures due to secondary and non-accidental trauma

Sugand et al., 2020 [28]	St Mary's Hospital, Chelsea (Level 1 trauma centre) and Westminster Hospital (Level 2 trauma unit), specialist tertiary centres for paediatric orthopaedic trauma and elective surgery), London, UK	All patients < 18 years with acute orthopaedic trauma presenting to the emergency department	2020/03/17–2020/04/28 vs. same period in 2019	Trauma referrals were reduced by two-thirds compared with 2019; greater use of outpatient telemedicine, with more virtual fracture clinic use; fewer patients seen for consultation and followed up face to face
Toy et al., 2021 [78]	Department of Orthopedics and Traumatology, Ağrı Training and Research Hospital, Ağrı, Turkey (rural region)	Admissions to orthopaedic and traumatology clinics with trauma (also adults)	2019/12–2020/08 grouped into 3-month periods: pre-covid (December, January and February), restriction period (March, April and May) and permitted period (June, July and August)	Significant reduction of admissions of children in restriction period
Verdoni et al., 2021 [29]	Orthopedic Trauma Hub Centre, Milan, Italy	Patients aged 0–16 admitted to the ER	2020/03/12–2020/05/05 vs. same period in 2019	Reduction of 87.0% in admissions; decreased with patient's age; trend towards more severe codes; fractures more frequent
Zacay et al., 2022 [31]	Electronic database of Meuhedet Health Services, which provides healthcare services to 1.2 million people in Israel	Patients < 18 years, fractures	2015–2020	Fracture rates were lower during all periods in 2020 than in the reference periods in 2015–2019; largest decline for age group 11–14 years
