# Supplementary material for: Influence of COVID-19 pandemic on hospitalisations at a paediatric traumatology department during 2020: a single-centre observational study and comprehensive literature review
Source: Eur J Trauma Emerg Surg. 2024 Jan 30;50(2):591–601. doi: 10.1007/s00068-024-02453-7 (PMC11035450; doi:10.1007/s00068-024-02453-7)
Supplement: Supplementary file 3 — Supplementary file3 (PDF 158 KB) [file 68_2024_2453_MOESM3_ESM.pdf]

Table 9 Results of studies regarding special injury types, locations or aetiologies (location or type of injury in bold)

Influence of COVID-19 pandemic in hospitalisations at a paediatric traumatology department during 2020: A single-centre observational study and comprehensive literature review

European Journal of Trauma and Emergency Surgery

Heide Delbrück*, Ellen Lambertz, Filippo Migliorini, Nina Berger, Frank Hildebrand

*Correspondence: hdelbrueck@ukaachen.de; ORCID 0000-0002-1676-4115

Author	Region	Considered patients	Periods	Main findings regarding pandemic period
Albright et al., 2022 [79]	National Electronic Injury Surveillance System (NEISS), emergency departments, US	All **hand fractures** in patients aged 1 to 19 years presenting to US EDs	2016–2020	39.2% decrease in the incidence of paediatric hand fractures; sports-related injuries 33% less likely
Auger et al., 2023 [58]	MED-ÉCHO, a registry of discharge abstracts for admissions throughout Quebec, Canada	**Cycling injury** hospitalisation	2006/04–2021/03	In March 2020, hospitalisation rates fell significantly for all age groups except children and adolescents.
Benmassaoud et al., 2022 [80]	Department of Pediatric Orthopedics and Traumatology, Hassan II University Hospital, Fez, Morocco	Children aged 0 to 15 years admitted for **domestic accidents**	2020/03/20–2020/07/10 vs. same period in 2019	Falls from heights of ≥ 2m most common accidents during lockdown (previous most common accidents in traffic)
Gornick et al., 2022 [44]	Leading trauma centre, freestanding children's hospital and paediatric-based tertiary referral centre, California, US	Children aged 0–18 years, surgically treated **fracture of the forearm, supracondylar humerus, femur or any other open fracture**	2020/03/01–2021/02/28 vs. 2018/03/01–2020/02/29	Decrease in injuries resulting from a fall from monkey bars for supracondylar humerus (21.2% to 8.2%, $p < 0.01$) and for forearm fractures (15.5% to 4.3%, $p = 0.04$); falls from a skateboard, hoverboard, scooter, bicycle and household furniture increased.

Gumina et al., 2021 [41]	Sapienza University of Rome, Umberto I Hospital Rome, Orthopaedics and Traumatology Unit, San Feliciano Hospital, Rome, Italy	Patients < 18 years, **shoulder and elbow trauma**	2020/03/08–2020/04/08 vs. same period in 2019	65% fewer first-aid shoulder/elbow services; paediatric patients for all types of injuries decreased by 84.6%; shoulder and elbow injuries mainly occurred because of accidental falls at home.
Gupta et al., 2021 [81]	Government Medical College and Hospital, Chandigarh, India	**Open fractures** (children and adults)	2020/03/25–2020/07/21 vs. same period in 2019	10 (19.23%) and 10 (11.23%), respectively (n.s.) paediatric patients
Heitz et al., 2023 [59]	CHU Sainte-Justine, Montréal QC, (urban, tertiary care, paediatric hospital), Canada	**Elbow fractures**	2020/04/01–2020/05/31 vs. same period 2016–2019	Fractures secondary to bicycle injuries were significantly higher in 2020, while falls from playground structures were lower.
Henry et al., 2022 [55]	Tertiary freestanding children's hospital, Children's Hospital of Philadelphia, US	Children < 2 years with skeletal surveys because of **concern about physical abuse**	2020/03/15–2020/10/15 vs. same period in 2019	22% decrease of skeletal surveys; no change in identification of occult fractures (6.9% pre-COVID vs 6.4% COVID, $p = 0.87$)
Johnson et al., 2021 [46]	Northeast, Southwest and West Level I paediatric trauma hospitals or associated paediatric orthopaedic surgery outpatient clinic sites; investigation performed at the Children's Hospital of Philadelphia, Pennsylvania, US	Common paediatric musculoskeletal **injuries associated with sports**	2020/03/15–2020/04/15 vs. same period in 2018 and 2019	The overall volume decreased; sports injuries decreased and the proportion of injuries occurring in the home increased.
Klepacki et al., 2022 [82]	Polish regional tertiary referral hospital	**Ankle joint injury**	2020/03/15–2020/10/20 vs. same period in 2019	Paediatric patient subpopulation showed a 70% decline (from 27 in 2019 to 8 in 2020, $p = 0.0423$); 1 surgery in 2020 vs. 0 surgeries in 2019
Köksal et al., 2022 [66]	Department of Orthopedics and Traumatology, Baltalimani Bone Diseases Training and Research Hospital, Istanbul, Turkey	**Tibial tubercle fractures (TTFs)**	2020/04–2020/05 vs. same period in 2019	Adolescent TTFs occurred during periods when outdoor activities were permitted (2019, 1 TTF/596 fractures, vs. 2020, 16 TTFs/504 fractures; patients were male and either overweight or at risk of being overweight.

Kovler et al., 2021 [52]	Johns Hopkins Children's Center in Baltimore, Maryland, US	Injuries caused by **physical child abuse (PCA)**	2020/03/28–2020/04/27 vs. same period in 2018 and 2019	Eight patients with PCA injuries were treated during the Covid-19 period (13% of total trauma patients) compared to four in 2019 (4%, $p < 0.05$) and three in 2018 (3%, $p < 0.05$).
Lãzãrescu et al., 2022 [53]	Necker Hospital for Sick Children, single regional paediatric neurosurgery centre, Paris metropolitan area, France	**Abusive head trauma (AHT)** in infants younger than 12 months	2017/01/01–2021/12/31	Marked increase in incidence and severity (mortality odds ratio, 9.39; 95% CI, 1.88–47.00) of AHT during the COVID-19 pandemic
McCauley et al., 2023 [54]	De-identified data obtained from the IBM Watson Health Explorys Cohort Discovery database, US	< 18 years with a new diagnosis of **nonaccidental trauma (NAT)**	2020 vs. 2019	Frequency of NAT remained unchanged; relative risk of fracture due to NAT was not significantly higher.
Payr et al., 2021 [20]	Level 1 trauma centre of the University Clinic of Orthopedics and Trauma Surgery in Vienna	All children aged 0 to 18 who presented with an injury	2020/03/16–2020/05/29 vs. same periods in 2015–2019	No significant decrease in fractures, but highly significant increase in **mild traumatic brain injuries**; incidence of wounds as a proportion of the total number of injuries increased; percentage of lacerations affecting the head compared to all lacerations was significantly higher.
Sabaghzadeh et al., 2022 [43]	Pediatric Orthopedic Trauma Center (Akhtar Public Hospital), Iran	**Supracondylar humerus fractures**; Gartland Type II and III	2020/02–2020/07 vs. same period in 2019	Transferring patients to the operating room was faster; surgeries of shorter duration; proportion of domestic injuries significantly increased.
Schultz et al., 2022 [83]	Large tertiary medical centre and its affiliated clinics, Vanderbilt University, Nashville, TN; Atrium Musculoskeletal Institute, Charlotte, NC, US	**Paediatric elbow fractures** (age 0–17)	2020/03–2021/03 vs. 2007–2017	Incidence in the same range but not associated with daylight or academic calendar; rate of fracture displacement was significantly elevated.
Shack et al., 2022 [60]	The Hospital for Sick Children in Toronto, Canada; large, urban tertiary referral centre for paediatrics and paediatric trauma	< 18 years, ED visit due to **bicycle-related injury**	2020/03–2020/10 vs. same period in 2018 and 2019	Significantly more bike injuries presenting to the ED per month;

Soriano et al., 2023 [42]	Department of Orthopedic Surgery, University of California, San Francisco, US (two tertiary children's hospitals)	< 12 years with surgery for a **supracondylar humerus fracture (SCH)**	2020/05–2020/11 vs. same period in 2019	SCH fractures decreased by > 50%; patients in 2020 were younger; injury mechanisms changed (more trampoline and furniture, less playground and monkey bars).
Springthorpe et al., 2022 [84]	Wythenshawe Hospital, Manchester Foundation Trust, Manchester, UK (two large teaching hospitals)	All orthopaedic **upper limb fracture** referrals, admissions and surgical procedures; also adults	2020/03/01–2021/02/28	Referrals, admissions and procedure rates for patients < 16 years decreased from March to June 2020.
Stivaros et al., 2022 [56]	Alder Hey Children's Hospital (Liverpool), Birmingham Children's Hospital, Bristol Children's Hospital, Leeds Children's Hospital, Nottingham University Hospitals NHS Trust, Pennine Acute Hospitals NHS Trust, Royal Manchester Children's Hospital and Sheffield Children's Hospital, UK	Children with **suspected physical abuse** (SPA, including abusive head trauma)	2018/01–2020/07	No difference between positive/negative radiographic skeletal surveys (SkS) rates
Theodorou et al., 2022 [51]	University of California Davis in Sacramento, CA, Lucile Packard Children's Hospital in Stanford, CA and Valley Children's Hospital in Madera, CA, US	< 18 years admitted after a **fall from a window**	2020/03/19–2020/09/19 vs. same period in 2019	Of 1,011 total COVID-era paediatric trauma patients, 36 (3.6%) sustained falls from windows compared to 23 of 1,108 (2.1%) pre-COVID era patients (OR 1.7, p = 0.05).

Walker et al., 2022 [85]	Department of Trauma and Orthopaedics, University Hospitals Dorset, Poole, UK	**Paediatric distal radial fractures**	2020/03/23–2020/07/03 vs. same period in 2019	194 fractures for the pre-COVID cohort and 101 fractures for the COVID cohort; no significant differences for any fracture pattern regarding age or sex; 40% of the pre-COVID cohort had a high mechanism of injury, compared with 66% of the COVID cohort ($p < 0.01$); significantly higher proportion of fractures were managed in a cast ($p < 0.001$); significantly fewer fractures managed in removable splints ($p < 0.001$); higher proportion of the COVID cohort underwent K-wire fixation ($p = 0.049$); COVID cohort had significantly more complications ($p = 0.016$) at a minimum 10-month follow-up.
Wild et al., 2022 [47]	Ann & Robert H. Lurie Children's Hospital, Chicago, IL, US	4–18 years, **acute sports or sports-type injury**	2020/03/20–2020/06/03 vs. same period in 2018 and 2019	Fewer sports injuries; patients were younger; frequency of surgical treatment was higher (more fractures).
