# Supplementary material for: Influence of COVID-19 pandemic on hospitalisations at a paediatric traumatology department during 2020: a single-centre observational study and comprehensive literature review
Source: Eur J Trauma Emerg Surg. 2024 Jan 30;50(2):591–601. doi: 10.1007/s00068-024-02453-7 (PMC11035450; doi:10.1007/s00068-024-02453-7)
Supplement: Supplementary file 4 — Supplementary file4 (PDF 126 KB) [file 68_2024_2453_MOESM4_ESM.pdf]

Table 10 Findings of studies regarding changed treatment processes

Influence of COVID-19 pandemic in hospitalisations at a paediatric traumatology department during 2020: A single-centre observational study and comprehensive literature review

European Journal of Trauma and Emergency Surgery

Heide Delbrück*, Ellen Lambertz, Filippo Migliorini, Nina Berger, Frank Hildebrand

*Correspondence: hdelbrueck@ukaachen.de; ORCID 0000-0002-1676-4115

Author	Region	Considered patients	Periods	Main findings regarding pandemic period
Fink et al., 2023 [63]	Trauma and Orthopedics, Gloucestershire Royal Hospital NHS Foundation Trust, Gloucester, GBR	Paediatric wrist and forearm fractures	2021/08/01–2022/01/31 vs. 2019/08/01–2020/01/31	Post COVID-19, 86.31% primary fracture manipulations in the ED following the guidelines in accordance with the BOAST recommendations for the early management of distal forearm fractures in children (improvement in comparison to 31.94% before the pandemic).
Hancock et al., 2021 [61]	Department of Trauma and Orthopedics, Sheffield Children's NHS Foundation Trust, Sheffield Children's Hospital, Sheffield, UK	Paediatric outpatient orthopaedics	2020/03/24–2020/05/10 vs. same period in 2019	Significant reduction in face-to-face appointments, radiographs per patient and discharge times; increase in telephone appointments and use of self-removable casting technique; no significant differences in complications related to casts or otherwise, or in unplanned attendance or reattendance after discharge; significant potential cost savings of > £185 000 per annum.
Johnson et al., 2022 [10]	Survey, electronically distributed to all POSNA members (147 respondents)	Surgeons caring for paediatric orthopaedic trauma patients were asked to respond.	Responses were collected from March 12 to April 9, 2021.	Outpatient fracture management: decreased number of follow-up visits for nondisplaced clavicle fractures, distal radius buckle fractures, and toddler's fractures; over 75% would continue these changed outpatient treatment schedules after the pandemic.
Memeo et al., 2020 [17]	Gaetano Pini Cto Hospital in Milan, Italy	Number of ER admissions, severity of the trauma and anatomical site	2020/02/23–2020/04/15 vs. same period in 2019	Reduction of 78% paediatric visits in the ER; rate of paediatric fractures increased by 21.62%; 11% of the total paediatric patients required orthopaedic surgery in 2020 (5.69% in 2019).

Payr et al., 2022 [49]	Department of Orthopedics and Trauma Surgery, Division of Trauma Surgery, Medical University of Vienna, Austria	Surgically treated paediatric upper limb fractures	2020/03/16–2020/05/29 vs. same period in 2015–2019	No difference in time until presentation, time until surgery and the length of stay at the hospital; number of complications documented did not increase; time until removal of implant was not significantly different; follow-up (appointments and radiographs) and clinical outcomes did not differ from the usual.
Verma et al., 2022 [30]	Trauma centre of King George's Medical University (KGMU), Lucknow, India	Patients up to age 18 admitted to the trauma centre	2019/10/01–2021/03/31	Higher times during the lockdown for time to the reception area at the trauma centre and time spent in the receiving area of the trauma centre; means ISS was significantly higher in the lockdown.
Zhu et al., 2023 [64]	The First and Second People’s Hospitals of Lianyungang, China, affiliated to Xuzhou Medical University	Douyin videos on paediatric humeral supracondylar fractures	Pre 2019 and post 2019	During the pandemic, views of videos regarding humeral supracondylar fractures increased.
